# SARS-CoV-2 and *Streptococcus pneumoniae* colonization and disease: an observational study in adults

**DOI:** 10.3389/fcimb.2025.1624521

**Published:** 2025-07-18

**Authors:** Sara Calvo-Silveria, Lucía Fernández-Delgado, Aida González-Díaz, Rocío España-Bonilla, Laura Calatayud, Jordi Niubó, Sara Martí, Ma Ángeles Domínguez, Jordi Càmara, Carmen Ardanuy

**Affiliations:** ^1^ Department of Microbiology, Hospital Universitari de Bellvitge – IDIBELL-UB, Barcelona, Spain; ^2^ Research Network for Respiratory Diseases (CIBERES), ISCIII, Madrid, Spain; ^3^ Department of Medicine, School of Medicine and Health Sciences, University of Barcelona, Barcelona, Spain; ^4^ Research Network for Infectious Diseases (CIBERINFC), ISCIII, Madrid, Spain; ^5^ Department of Pathology and Experimental Therapeutics, University of Barcelona, Barcelona, Spain

**Keywords:** *Streptococcus pneumoniae*, serotypes, SARS-CoV-2, COVID-19, co-infection

## Abstract

**Introduction:**

The COVID-19 pandemic has impacted global health and altered respiratory pathogens. While SARS-CoV-2 vaccines have mitigated COVID-19 severity, emerging variants remain challenging. Co-infection of *Streptococcus pneumoniae* with respiratory viruses is associated with increased disease severity, but its relationship with SARS-CoV-2 remains unclear. This study aims to analyze their co-occurrence, focusing on disease progression, colonization rates and clinical outcomes.

**Methods:**

To this end, three approaches were used. First, a laboratory-based analysis of invasive pneumococcal disease (IPD) in adults (2019-2023). Second, a retrospective analysis of COVID-19 clinical cases with pneumococcal isolates (March,2020–December,2023), including clinical and microbiological data such as patients’ comorbidities, episode severity, serotypes and resistance genes. Third, a retrospective analysis to assess pneumococcal colonization in SARS-CoV-2 positive nasopharyngeal samples (May-October 2023; dual-target RT-PCR). WGS and bioinformatics were performed on both bacterial (serotyping and resistance analysis) and viral genomes (lineage determination). Statistical comparisons (Chi-square, Fisher’s test), with significance set at *p*<0.05.

**Results:**

First, IPD incidence declined during the COVID-19 pandemic, with cases dropping by 70% in both age groups (18–64 and >64) from 2019 to 2021 and rebounding after 2021, concomitant with the relaxation of non-pharmaceutical measures, especially among older adults. Pneumococcal serotype distribution remained stable with dominance of serotypes 3 and 8. Serotype 12F disappeared during the lockdown and re-emerged in 2023 as a multidrug-resistant sub-lineage through multi-fragment recombination, derived from the former GPSC26. Second, SARS-CoV-2 and pneumococcal co-infection occurred in 66 hospitalized patients, mainly by serotype 3 (15%), with resistance to macrolides (26.3%) and tetracycline (22.8%). Third, pneumococcal colonization in SARS-CoV-2-infected patients was low (2.8%), especially in older adults (>64 years; 1.5%), with slightly higher rates in severe cases (4.7% vs 2.5%; *p*=0.404; IC95% 0.13-3.05) and young adults (4.8% vs 1.5%; *p*=0.04; IC95% 0.92-15.21). Compared to colonized patients, those with co-infection had more comorbidities, more severe clinical presentations, higher hospitalization rates and lower vaccination rates.

**Discussion:**

This study highlights how non-pharmaceutical measures disrupt *S. pneumoniae* dynamics. Although pneumococcal colonization in SARS-CoV-2 patients appears to be infrequent, our data suggest an increase in disease severity. Then, vaccination programs and their monitoring remain critical in the prevention of respiratory infections.

## 1 Introduction

The coronavirus disease (COVID-19) pandemic emerged as one of the most profound global health crises of the modern era. Since its identification in late 2019, SARS-CoV-2 has led to over 400 million confirmed cases and approximately 6 million deaths worldwide (covid19.who.int/, last accessed 2025-02-25). The rapid development and deployment of SARS-CoV-2 vaccines in late 2020 marked a turning point in controlling severe disease and mortality, particularly in high-income countries with extensive vaccination coverage (Rinott et al., 2020). However, the ongoing emergence of SARS-CoV-2 variants, characterized by partial escape from vaccine-induced immunity, continues to challenge global efforts (Garcia-Beltran et al., 2021).

In addition to its direct effects on morbidity and mortality, the pandemic has had substantial indirect consequences on the epidemiology of other infectious diseases. COVID-19 containment measures, including lockdowns, social distancing, mask-wearing, and enhanced hygiene practices, have significantly altered the transmission dynamics of other respiratory pathogens. Studies have reported marked declines in the incidence of bacterial agents causing respiratory infections, as well as seasonal viruses such as influenza and respiratory syncytial virus (Brueggemann et al., 2021; Doroshenko et al., 2021; Guisado-Gil et al., 2022). These reductions may reflect a combination of factors, including the COVID-19 containment measures and a potential underreporting due to the overwhelming focus on COVID-19 diagnostics and surveillance (Dirkx et al., 2021).

The interplay between respiratory viruses and bacterial pathogens has been largely documented, particularly in the context of co-infections. *Streptococcus pneumoniae* is the most common bacterial pathogen associated with community acquired respiratory tract infections and invasive diseases (IPD). The nasopharynx serves as the primary reservoir for pneumococcal colonization, especially in young children, although adults may also carry pneumococci at a lower frequency (Weiser et al., 2018). Pneumococcal infections are frequently preceded by viral infections, which can disrupt the epithelial barrier, alter lung physiology, and promote bacterial colonization. Influenza virus, for instance, is known to facilitate pneumococcal adherence, replication, and dissemination, leading to severe co-infections and poor clinical outcomes (Kim et al., 1996; McCullers, 2014; Mina et al., 2014; Lewnard et al., 2019; Manna et al., 2022). Given these established relationships, it is plausible that SARS-CoV-2 could similarly influence pneumococcal colonization and infection. However, data on the relationship between SARS-CoV-2 and *S. pneumoniae* remains scarce. Studies analyzing the frequency of co-infection in hospitalized COVID-19 patients have reported variable rates depending on the diagnostic methodologies and study populations (Soto et al., 2021; Cohen et al., 2022). Notably, polymerase chain reaction (PCR) based methods have demonstrated higher sensitivity for detecting pneumococcal colonization compared to traditional culture techniques, which are often limited in patients receiving antibiotics (Zacharioudakis et al., 2021). In addition, the clinical impact of SARS-CoV-2 and *S. pneumoniae* co-infection remain inconclusive, with some studies suggesting minimal impact on patient outcomes, while experimental data indicate potential immune modulation during co-infection (Jochems et al., 2019; Rothe et al., 2021).

The potential relationship between SARS-CoV-2 and *S. pneumoniae* underscores the importance of ongoing surveillance. Understanding the dynamics is critical not only for managing co-infections but also for anticipating the broader implications of viral-bacterial associations. In this study, we analyzed the correlations between SARS-CoV-2 and *S. pneumoniae* in disease and colonization.

## 2 Materials and methods

### 2.1 Study design

To achieve the goals of this study, three different approaches were conducted at Hospital Universitari de Bellvitge (HUB), a teaching hospital located in the southern Barcelona area. The Microbiology Service receives samples for diagnosis from primary care centers and from patients who come to the hospital (**Supplementary Figure S1**).

The first approach was to analyze changes in invasive pneumococcal diseases (IPD) during the COVID-19 pandemic. All IPD episodes in adults (≥18 years old) from January 2019 to December 2023 were retrospectively collected and available pneumococcal isolates were subjected to antibiotic susceptibility testing and WGS. IPD was defined as the isolation from a sterile body site in a patient with signs and symptoms of infection. To contextualize the monthly trends in IPD including the pandemic period, data from 2010 to 2023 was analyzed.

The second approach was to investigate the relationship between pneumococcal disease and COVID-19. All patients with SARS-CoV-2 who had a *S. pneumoniae* isolate from a clinical sample between March 2020 and December 2023 were included (n = 66). Available pneumococcal isolates were sequenced (WGS, n= 57) and clinical charts were reviewed.

Third approach was to assess *S. pneumoniae* colonization in patients with SARS-CoV-2. A random selection of SARS-CoV-2 positive nasopharyngeal samples (routinely diagnostic procedures) of patients were collected between May and October 2023 (n = 461). A third of the samples were collected from patients attending primary care centers and the rest from hospital patients (only one sample per patient was included). COVID-19 disease was defined as the detection of SARS-CoV-2 in a patient presenting symptoms of infection such as cough, fever, shortness of breath, sudden onset of anosmia, ageusia or dysgeusia. Patients that did not require further hospitalization were considered mild cases while those requiring hospital care were classified as severe. All samples were prospectively stored at -80°C for pneumococcal colonization detection by RT-PCR and viral genome sequencing. Clinical charts were reviewed for the positive pneumococcal colonization patients and representative negative control group.

### 2.2 Characterization of pneumococcal isolates

The identification of *S. pneumoniae* isolates was routinely performed using standard microbiological procedures (MALDI-Biotyper, bile solubility and/or optochin susceptibility). Antibiotic susceptibility was determined by disk diffusion and E-test in accordance with EUCAST recommendations and criteria.

### 2.3 Nasopharyngeal colonization analysis

Pneumococcal colonization was studied using a dual-target RT-PCR assay (*lyt*A and *pia*B genes) in nasopharyngeal samples. Colonization was confirmed when both targets were detected and the cycle threshold (Ct) values were similar (differences <3 Ct) (Simões et al., 2016; Miellet et al., 2023).

Nasopharyngeal samples were considered positive for SARS-CoV-2 if detected by RT-PCR with a Ct value <37 (Alinity m Resp-4-plex assay) (S. T. Almeida et al., 2020).

### 2.4 Bacterial genome sequencing and bioinformatic analysis

For library preparation and sequencing, *S. pneumoniae* was cultured overnight on 5% sheep blood agar at 37°C with 5% CO2. DNA was extracted using the QIAamp DNA Mini Kit (Qiagen, Germany) and quantified with the Qubit dsDNA HS Assay Kit (Thermo Fisher, USA). Illumina paired-end libraries (2x300 bp) were prepared using the DNA Prep kit and sequenced on the Illumina MiSeq Platform (Illumina, USA).

For bioinformatic analysis, quality assessment and genome assembly were performed using the Bactopia pipeline. Reads quality control was conducted with Bactopia’s preprocessing module, and genome assembly was performed using the assembly module. The Bactopia MLST module was used for *in silico* MLST determination using PubMLST database (web PubMLST). In silico serotyping was performed with SeroBA (github.com/sanger-pathogens/seroba) (**Supplementary Table S1**). The reads were deposited in the European Nucleotide Archive (ENA), and the metadata is outlined in **Supplementary Table S2**. Antibiotic resistance due to mutations in resistance-related genes, such as *pbp*1a, *pbp*2b and *pbp*2x (β-lactams), *par*C, *par*E and *gyr*A (quinolones), or *fol*A and *fol*P (cotrimoxazole), was analyzed and compared with the reference genome (*S. pneumoniae* R6, NC_003098.1) using Geneious. Acquired resistance genes were identified using AMRfinder from Bactopia workflow.

For in-depth analysis of serotype 12F recombination events, 14 genomes from pneumococci isolated in our hospital between 2008 and 2023 were selected. Recombinant blocks were detected by Gubbins using NZ_LS483450 as reference and default parameters.

### 2.5 Viral genome sequencing and bioinformatic analysis

Genomic sequencing of SARS-CoV-2 was performed following the ARTIC amplicon sequencing protocol (artic.network/ncov-2019).Total nucleic acid extraction was performed with MagMAX Viral/Pathogen II Nucleic Acid Isolation Kit on a KingFisher Flex purification system (Applied Biosystems, USA). The Ct was determined using the TaqPath COVID-19 RT-PCR assay on a QuantStudio 5 (Applied Biosystems, USA), and only samples with a Ct value <30 were sequenced. RNA retrotranscription was carried out with LunaScript (New England BioLabs, USA), followed by amplification of the 30 kb viral genome using the xGen SARS-CoV-2 ARTIC Amplicon Panel (v4.1), which generates 400 bp amplicons (IDT, USA). DNA quantification was performed using the Qubit dsDNA HS Assay Kit (Thermo Fisher, USA). For library preparation and sequencing, Illumina paired-end libraries (2x200 bp) were prepared using the Illumina DNA Prep kit and sequenced on the Illumina MiSeq Platform (Illumina, USA).

Bioinformatic analysis was conducted using the DRAGEN COVID Lineage App from BaseSpace (basespace.illumina.com). Pangolin was used to assign lineages to the COVID-19 sequences. Consensus genomes and demographic data were deposited at the GISAD web page (https://www.gisaid.org) for public access.

### 2.6 Statistics

*Chi*-square test and Fisher’s exact test were applied to assess the association between categorical variables, set at α < 0.05 (two-tailed) for significance. Chi-square test was applied by default, while Fisher’s exact test was applied when the expected frequency in any cell was less than 5. Tests were performed using the chisq.test and fisher.test functions in R (version 4.3.2), respectively. To analyze changes in the serotype and clonal distributions of invasive isolates, three periods were considered: pre-pandemic (January 2019-March 2020), pandemic (April 2020-June 2022), and post-pandemic (July 2022-December 2023).

## 3 Results

### 3.1 Invasive pneumococcal disease decreased during the SARS-CoV-2 pandemic and upsurged after relaxation of non-pharmaceutical measures

**Figure 1:**
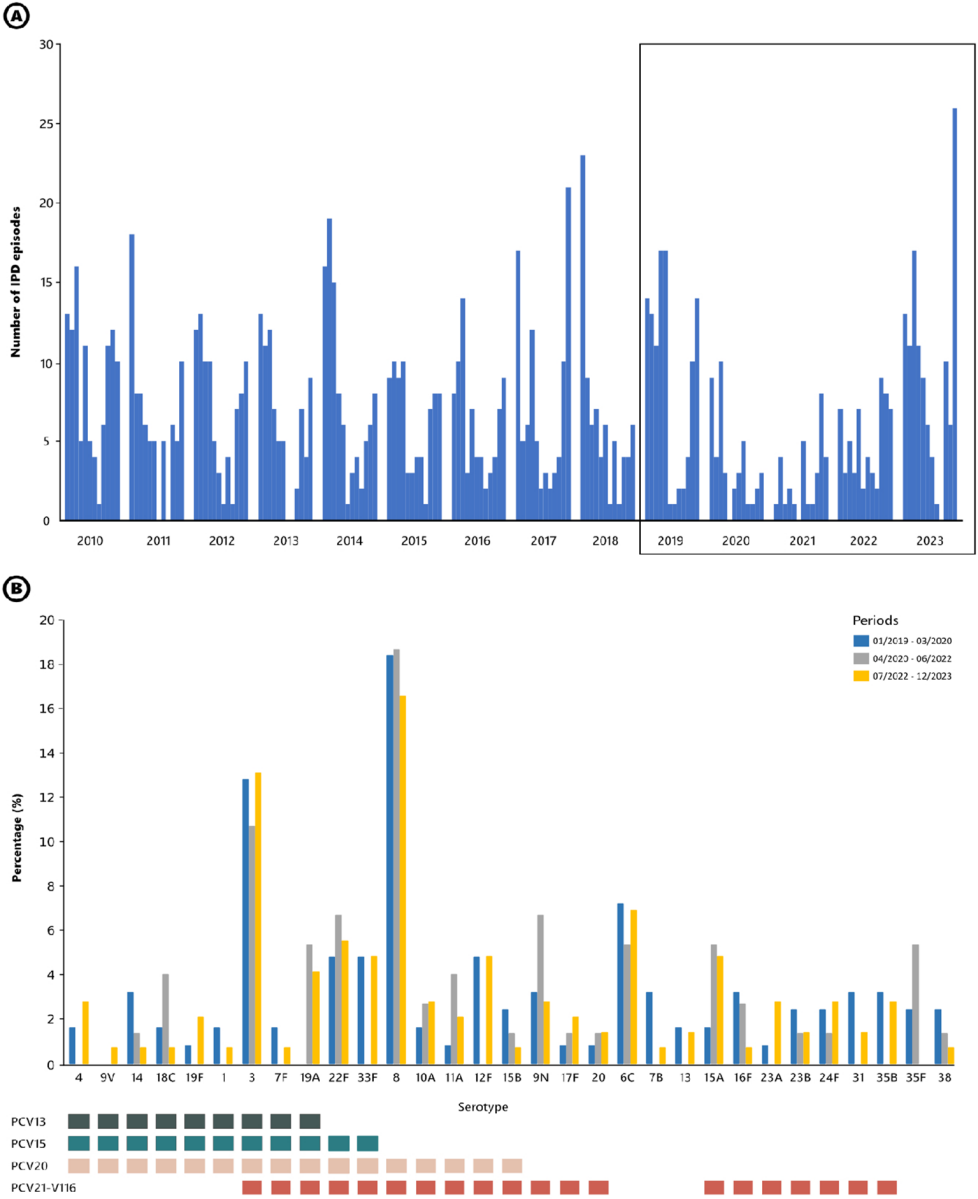
**(A)** Monthly occurrence of IPD episodes in adult patients attended at Hospital Universitari de Bellvitge from 2010 to 2023. The boxed section highlights the period from 2019 to 2023. **(B)** Serotypes causing IPD before, during, and after the SARS-CoV-2 pandemic and lockdown. Bars represent the frequency of each serotype during the period analyzed (corresponding to the boxed section in panel **(A)**. Squares below highlight the serotypes included in the different vaccine formulations. All vaccines have been approved by EMA and FDA.

From January 2019 to December 2023, a total of 355 IPD episodes were recorded. Of these, 150 episodes occurred in adult patients aged 18 to 64 years and 205 in those over 64 years old. The monthly number of IPD episodes detected from 2010 to 2023 is shown in **Figure 1A**. The seasonal fluctuation of IPD was interrupted during the pandemic, with a sharp decrease in the number of IPD episodes (from 105 episodes in 2019 to 44 in 2020 and 31 in 2021). The lockdown due to the SARS-CoV-2 pandemic began in Spain in March 2020. Following this, the number of IPD episodes decreased, with no winter season peak during 2020-2021 (n = 11). Subsequently, winter seasons were characterized by a limited recovery in IPD numbers during 2021-2022 (n = 30) and 2022-2023 (n = 65).

The decline and recovery of IPD cases varied by age group (**Supplementary Figure S2**). In the 18–64 age group, there was a sharp decline from 2019 to 2021, representing a decrease of 68.3%. Recovery began in 2022 but rebounded significantly in 2023 with a total number exceeding the pre-pandemic levels (+26.8%). For patients over 64 years old, the decline was even more evident and pronounced. Cases dropped from 2019 to 2021 (70.6% reduction). After limited recovery in 2022, the figure at the end of 2023 was similar to 2019 (–6.2%).

To identify resilient serotypes during the lockdown (**Figure 1B**), we analyzed the serotype distribution of IPD episodes. We compared three periods: pre-pandemic (January 2019-March 2020), pandemic (April 2020-June 2022), and post-pandemic (July 2022-December 2023). Overall, the serotype distribution remained stable across all three periods, with predominance of serotypes 3 and 8. Notably, although the numbers were low, serotype 12F disappeared during the lockdown, while serotype 9N emerged. The proportion of IPD cases covered by PCV15, PCV20 and PCV21 in the three periods was: 32.8%, 60.8% and 64.0% in the pre-COVID period; 28.0%, 54.7% and 61.3% during the COVID-19; and 35.9%, 62.8% and 65.5% in the post-COVID period, respectively.

**Figure 2:**
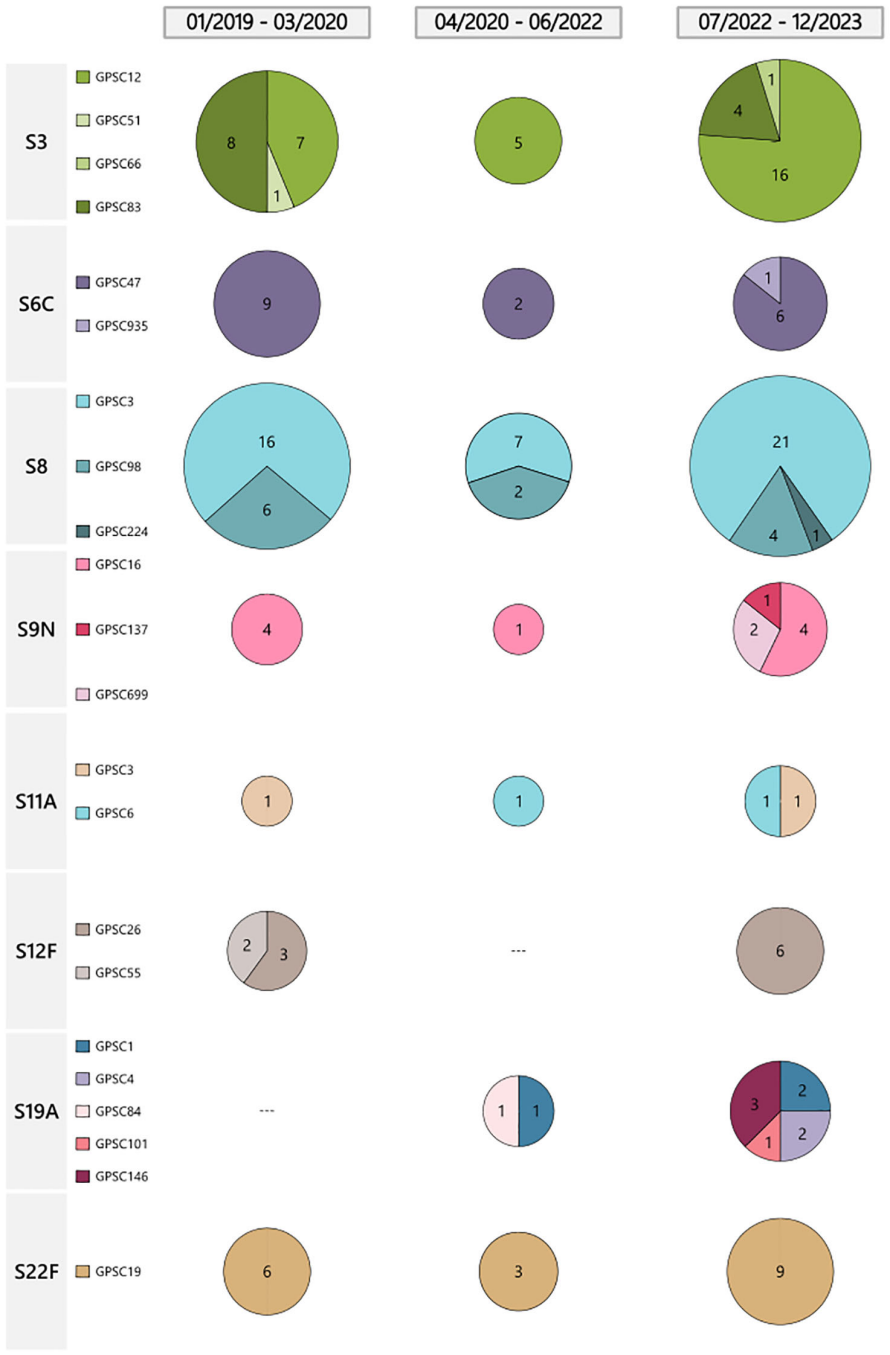
Distribution of GPSC of major serotypes causing IPD by period. Pie charts represent the number of isolates for each Global Pneumococcal Sequence Cluster (GPSC) of the most prevalent serotypes. Each serotype (S) contains different GPSCs, each one represented by a distinct color within the pie charts. The size of the pie is proportional to the number of isolates.

Regarding the Global Pneumococcal Sequencing Cluster (GPSC) and Sequence Type (ST) distribution of the main serotypes, different patterns were observed. For example, GPSC3-ST53 was predominant in serotype 8 isolates across all periods (**Figure 2**). Among serotype 3 isolates, GPSC12-ST180 was nearly as prevalent as GPSC83-ST260 before the pandemic, and it became clearly dominant in the post-pandemic period. Regarding serotype 12F, a notable change was observed. Before the pandemic, this serotype consisted of GPSC26-ST989 (n = 3) and GPSC55-ST8060 (n = 2). This serotype was not detected during the pandemic and re-emerged in the post-pandemic period in association with a new sub-lineage of GPSC26-ST3377 exhibiting a multidrug resistant phenotype (MDR, penicillin and macrolide resistance, see below). Among serotype 9N isolates, genetic diversification was observed. During the first two periods, including part of the pandemic, this serotype was exclusively composed of GPSC16-ST66. In the post-pandemic, the number of cases increased, and this serotype was represented by three GPSCs: GPSC16-ST66 (n = 4), GPSC699-ST19827 (n = 2), and GPSC137-ST3982 (n = 1).

### 3.2 Characterization of pneumococcal strains from COVID-19 patients

During the SARS-CoV-2 pandemic (March 2020 to December 2023), 66 COVID-19 patients admitted to the hospital had concomitant pneumococcal infection (**Supplementary Table S3**). A total of 52 episodes were classified as non-invasive, while 14 were considered IPD. The most common serotypes (57/66 serotypes available) were 3 (17.5%), 11A (15.8%), 6C (8.8%), 23A (8.8%), and 19F (5.3%). Despite the relatively low numbers of episodes per year, notable changes in the prevalence of certain serotypes coinfecting SARS-CoV-2 patients were observed. For example, serotype 3 was consistently present in 2020 (10.0%), 2021 (18.2%), and 2022 (30.4%), but it was not detected in 2023 (**Supplementary Figure S3**). In contrast, serotypes 11A and 6C were detected in all four years.

**Figure 3:**
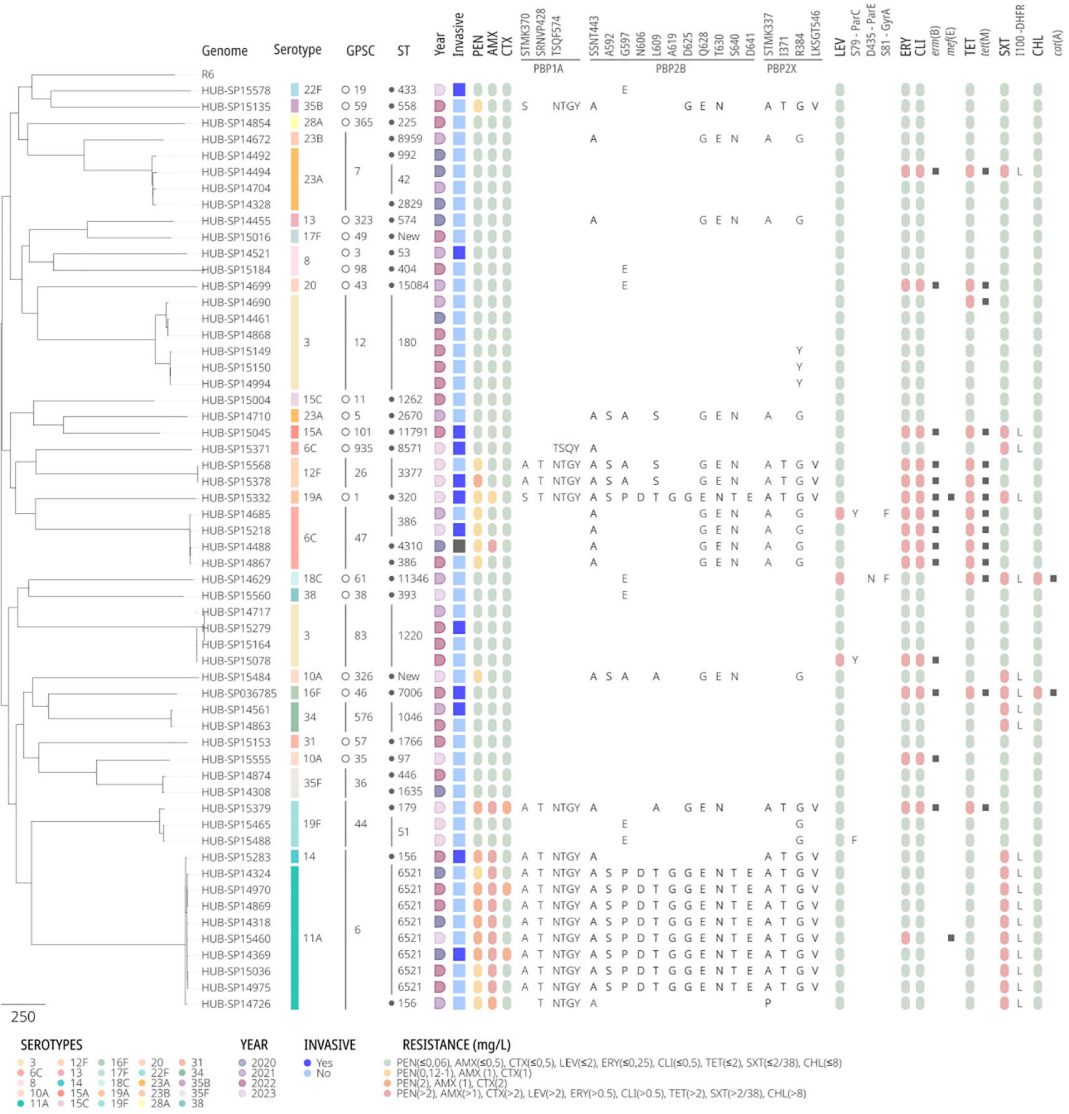
Phylogenetic tree of pneumococcal strains causing infection in COVID-19 patients. Each row represents a single isolate. The columns represent a heatmap with the presence/absence of either phenotypic (resistance/susceptibility) or genotypic traits analyzed *in silico*. The genomic analysis includes typing: serotype, Global Pneumococcal Sequencing Cluster (GPSC), Sequence Type (ST); AA changes at the transpeptidase domain of PBPs, quinolone resistance determining regions or DHFR; acquired antimicrobial resistance genes. Antibiotics are abbreviated as follows: PEN, penicillin; AMX, amoxicillin; CTX, ceftriaxone; LEV, levofloxacin; ERY, erythromycin; CLI, clindamycin; TET, tetracycline; SXT, sulfamethoxazole-trimethoprim; and CHL, chloramphenicol.

Given the high use of antibiotics during the SARS-CoV-2 pandemic, especially in the early stages, we analyzed resistance rates among the *S. pneumoniae* isolates from COVID-19 patients (**Figure 3**). All 57 sequenced isolates had penicillin MICs ≤ 2 mg/L (range ≤0.06–2 mg/L). Fifteen isolates (26.3%) were macrolide-resistant, harboring *erm*(B) and/or *mef*(E) genes. Tetracycline-resistance (n = 13, 22.8%) was associated with the presence of the *tet*(M) gene. The resistance rates for co-trimoxazole and levofloxacin were 15.8% and 5.3%, respectively. In both cases, the resistance was linked to point mutations in target genes. **Figure 3** displays the phylogenetic tree of pneumococci isolated from COVID-19 patients.

**Figure 4:**
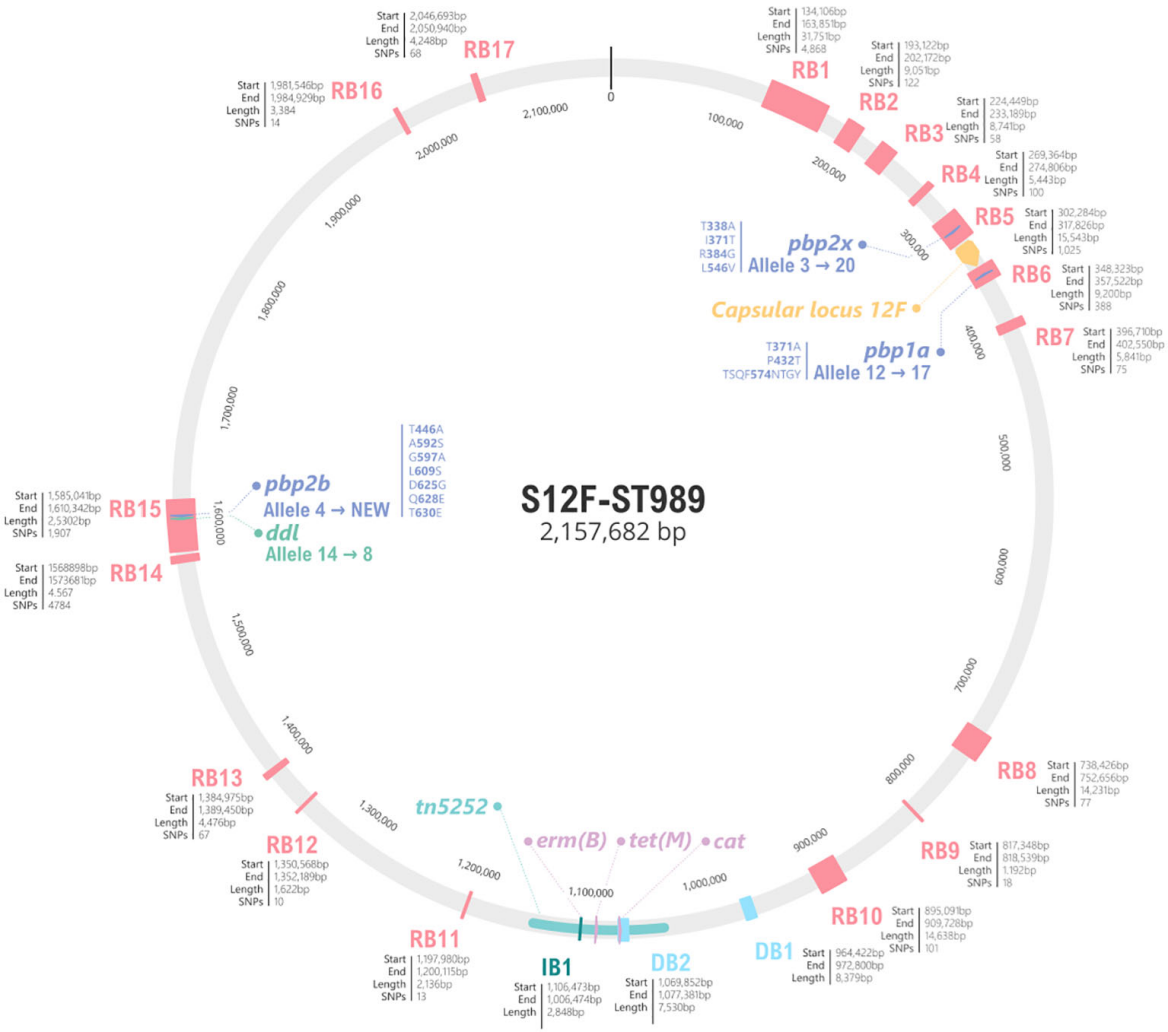
Schematic representation of genomic differences between serotype 12F ST989 and ST3377 of GPSC26. The grey circle represents the genome of NZ_LS483450 (ST989 serotype 12F), in pink the 17 recombination blocks detected by Gubbins. For each block, the start, the end and the total length are specified. For PBP2X, PBP1A and PBP2B amino acid changes involve in beta-lactam resistance are specified. The capsular locus is marked in yellow and antibiotic resistance genes in purple. Genome fragments not present in ST3377 are marked in light blue as DB (deletion block), on the other hand, genome acquisitions are represented in dark blue, as IB (insertion block).

The re-emergence of serotype 12F in 2023 was associated with a new ST, the ST3377, of the major GPSC26 lineage. This sub-lineage exhibited MDR phenotype, including non-susceptibility to penicillin, macrolides, clindamycin, and tetracycline. After a deeper analysis (**Figure 4**; **Supplementary Table S4**), a total of 17 recombination blocks (RB) between ST989 and ST3377 were detected. Some of these RB included important genes such as *pbp2x* in RB5 that changed from allele 3 in ST989 to 20 in ST3377, *pbp1a* in RB6 which changed from allele 12 to 17 or *pbp2b* (allele 4 to new) in RB15. This new PBP type in ST3377 results in an increase of penicillin (1 mg/L) and amoxicillin (2 mg/L) MICs. RB15 also includes *ddl* gene explaining the ST change in this new sub-lineage. In addition, the different rearrangements of the Tn5252 present in the reference genome include the loss of the *cat* gene (resistance to chloramphenicol) and the acquisition of the *erm*(B) which confers resistance to erythromycin and clindamycin. There were no differences in the structure of the capsular operon between strains of these two sub-lineages. This acquired resistance represents a new hallmark of serotype 12F and warrants close surveillance.

### 3.3 The rate of pneumococcal colonization in patients with COVID-19 disease is low

A total of 461 nasopharyngeal swabs from patients with SARS-CoV-2 infection, who attended primary care centers (n = 133) or the hospital (n = 328), were analyzed (**Table 1**). Hospitalized patients were also tested for influenza viruses (A and B) and respiratory syncytial virus (RSV), but none tested positive, indicating no viral co-detection. Pneumococcal colonization was screened through a dual-targeted PCR (*lyt*A plus *pia*B). Of the 461 samples studied, 181 (39.3%) were from men and 273 (59.2%) from patients over 64 years of age. Pneumococcal colonization was detected in 13 patients (2.8%), 10 of whom presented mild COVID symptoms. In concordance with the circulating SARS-CoV-2 variants in the period studied, the majority of infections, both in colonized and non-colonized patients, involved recombinant XBB variants. **Table 1** presents the analysis of pneumococcal colonization in patients with SARS-CoV-2 infection. The colonization rate was similar in primary care (2.3%) and hospitalized (3.0%) patients. In terms of disease severity, colonization was more common in patients with severe COVID-19 (4.7%) compared to those with mild disease (2.5%) (*p* = 0.404; IC 95% 0.13-3.05). By age group, the colonization rate was higher in young adults (18–64 years old, 4.8%) than in older people (>64 years old, 1.5%) (*p* = 0.04; IC 95% 0.92-15.21). Regarding differences in colonization among Omicron lineages, some slight variations were observed although they did not reach the statistical significance. Further statistical analysis adjusting for confounding factors was not possible due to low colonization rates.

**Table 1:** Pneumococcal colonization among patients with SARS-CoV-2 infection.

	Number of samples tested	*S. pneumoniae* positivity
Overall	461	13 (2.8%)
Source		
Primary care	133	3 (2.3%)
Hospital	328	10 (3.0%)
Disease severity		
Mild	397	10 (2.5%)
Severe	64	3 (4.7%)
Age		
18-64 years	188	9 (4.8%)
>64 years	273	4 (1.5%)
SARS-CoV-2 variant		
XBB	266	9 (3.4%)
EG	62	0 (0%)
FL	19	1 (5.3%)
FG	16	1 (6.3%)
JG	16	0 (0%)
Other	71	2 (2.4%)

### 3.4 Clinical characteristics of patients with pneumococcal colonization or infection in SARS-CoV-2 patients

We compared the clinical characteristics of SARS-CoV-2 patients between two groups: patients with SARS-CoV-2 and pneumococcal colonization (n = 10, 3 clinical charts unavailable) versus patients with SARS-CoV-2 and pneumococcal infection (n = 66) (**Table 2**). We also selected a control group of patients with only SARS-CoV-2 infection (n = 26). In general, patients without pneumococcal colonization or infection had fewer underlying conditions and comorbidities compared to those with pneumococcal colonization. The clinical presentation of patients with pneumococcal infection was more severe, with a higher rate of pneumonia (65.2%) and respiratory failure (69.7%). These patients also required hospital admission more frequently (84.8%).

**Table 2:** Characteristics of patients with SARS-CoV-2 infection regarding pneumococcal colonization/infection.

	Pneumococcal infection (n=66), number of episodes (%)	Pneumococcal colonization- (n=10), number of episodes (%)	Non-pneumococcal colonization (n=26), number of episodes (%)
Age (years). mean (SD)	68.9 (14)	53.5 (22.6)	53.2 (24)
Female	14.9 (22.6)	7 (70)	13 (50)
Underlying conditions			
Current or former smoker	40 (60.6)	8 (80)	7 (26.92)
None comorbidities	10 (15.2)	3 (30)	14 (53.85)
2 or more comorbidities	47 (71.2)	6 (60)	7 (26.92)
Clinical presentation			
Pneumonia	43 (65.2)	2 (20)	3 (11.54)
Other respiratory infections	23 (34.8)	8 (80)	23 (88.46)
Severity and outcome			
Sepsis	13 (19.7)	0 (0)	0 (0)
Respiratory failure	46 (69.7)	3 (30)	2 (7.69)
Hospital admission	56 (84.8)	3 (30)	4 (15.38)
ICU admission	15 (22.7)	0 (0)	1 (3.85)
30-day mortality	11 (16.7)	0 (0)	0 (0)
Vaccionation history			
SARS-CoV-2	37 (56.1)	10 (100)	22 (84.62)
Influenza A	34 (51.5)	9 (90)	13 (50)
PCV13	7 (10.6)	2 (20)	3 (11.54)
PCV20	1 (1.5)	2 (20)	5 (19.23)
PPV23	5 (7.6)	4 (40)	8 (30.77)

The rate of pneumococcal vaccination was low in patients with pneumococcal infection. Almost all patients were appropriately vaccinated against SARS-CoV-2. However, as some pneumococcal co-infection episodes occurred before SARS-CoV-2 vaccines were available, the vaccination rate in this group was lower. To address this, we analyzed this group in more detail, differentiating between episodes occurring before SARS-CoV-2 vaccines were available (prior to February 2021) and those after vaccines became available (from February 2021 onward). Patients prior to February 2021 had more risk factors and comorbidities, with half of them having more than two comorbidities, compared to 15 out of 55 (27.3%) in the other group. Their clinical presentation was also more severe, and their 30-day mortality rate was higher (27.3% vs 6.5%). Pneumococcal serotypes also varied between the groups: serotype 3 was nearly absent in the pre-vaccine period, whereas it was the most prevalent after February 2021. There were only two cases of pneumococcal infection in PCV13-vaccinated patients, with strains belonging to serotype 3 and 19F.

## 4 Discussion

In this manuscript, we explored the associations between SARS-CoV-2 and *S. pneumoniae* over the three pandemic years. During the COVID-19 pandemic, IPD drastically decreased, likely due to non-pharmaceutical interventions (NPIs), such as mask usage, social distancing, and lockdowns. These measures not only limited SARS-CoV-2 transmission but also disrupted the circulation of other respiratory viruses and bacterial pathogens, including pneumococcus (Brueggemann et al., 2021).

Respiratory viral infections are known to predispose individuals to bacterial infections by promoting bacterial shedding and disrupting the integrity of the respiratory epithelium (Howard, 2021; Mitsi et al., 2024). It has also been described that bacterial colonization in patients with viral infection could enhance the severity of the disease (Rodriguez-Fernandez et al., 2023). Viral infection causes damages to the respiratory cells, facilitating the invasion and colonization by bacterial pathogens. Additionally, vascular permeability in the location is increased in response to damage, promoting the access of bacteria to the alveoli (Fazel et al., 2023). Our data indicate a low prevalence of pneumococcal colonization in patients with SARS-CoV-2 during the pandemic, similar to rates described in studies analyzing pneumococcal colonization in adults. This is also consistent with reports from other studies that showed low bacterial co-infections rates in COVID-19 patients. For example, a retrospective cohort study identified pneumococcal colonization in only 1.1% hospitalized COVID-19 patients (Coenen et al., 2021). Similarly, a recent study on COVID-19 pneumonia found a 7.5% co-infection rate, underscoring that bacterial co-infections are infrequent (Strelkova et al., 2022). It seems that social distancing and the high use of antibiotics during the pandemic may have affected pneumococcal transmission as well as other respiratory pathogens. Nevertheless, the colonization rate in SARS-CoV-2 infected patients should be revisited in a few years, once viral dynamics have stabilized.

IPD cases progressively increased following the relaxation of NPIs. By December 2023, the total number of IPD episodes was the highest recorded in the last 13 years in our institution, coinciding with the resurgence of other respiratory viruses, such as RSV and influenza. During the 2022–2023 winter season, RSV predominantly affected the pediatric population, while adult IPD cases approached pre-pandemic levels. The subsequent 2023–2024 winter season saw a sharp increase in influenza virus, which was associated with a significant peak in IPD cases (Sender et al., 2021). These data highlight the close relationship between respiratory viruses and pneumococcal diseases (Manna et al., 2022). Nevertheless, the figure of IPD for adults over 65 was lower than in pre-pandemic years. It is possible that some lessons learned, such as maintaining social distance from older adults or individuals with comorbidities in cases of respiratory illness, have persisted after the pandemic. However, in early 2023 there was a change in the official strategy for prevention of pneumococcal diseases in our area, introducing PCV20 for adults over 65 and PCV15 for children. Both factors could have contributed to this decline. The introduction of these new PCV is expected to decrease pneumococcal colonization across a broader group of serotypes but also in a new group of age, as older adults were previously vaccinated with polysaccharide vaccines that do not prevent colonization. The high fraction of serotypes covered by these and other available vaccines highlight the potential benefit of adult vaccination in reducing the burden of disease. Adult vaccination will likely offer additional benefits to this population. For instance, some studies suggested that pneumococcal vaccination may have broader effects, including a reduction in hospitalizations caused by endemic coronaviruses and outcomes related to COVID-19 in older adults (Mitsi et al., 2022). Also, pneumococcal colonization has been reported to diminish inflammatory response to viral infections, leading to more severe outcomes (Mitsi et al., 2024). However, this effect on disease severity has not yet been demonstrated in SARS-CoV-2 infections, but potentially it could be prevented by PCV vaccination. Nevertheless, the potential emergence of new pneumococcal lineages warrants further surveillance to understand their role in post-pandemic IPD trends.

The pandemic highlighted the crucial role of vaccination programs in preventing infectious diseases, reducing most vaccinable serotypes. However, we detected a resurgence of some serotypes included in PCV7 and PCV13, such as serotype 19A. Furthermore, serotype 3 levels remained high throughout the whole period, and serotypes 4 and 18C showed a slight increase. Several factors could explain these trends. First, young adults who were not vaccinated during childhood are now reaching ages where they are more susceptible to pneumococcal infections. Second, older adults and people with comorbidities were mostly vaccinated with polysaccharide vaccine as mentioned before. Third, the subsequent introduction of new serotypes in the vaccine formulations aims to reduce IPD caused by those serotypes. However, their impact on colonization is expected to decrease, as some of the newly included serotypes have shown lower immunogenicity.

It is interesting to note the genetic background of pneumococcal serotypes after the pandemic. First, the success of GPSC3-ST53 (serotype 8), which confirms the high invasive disease potential of this lineage (Hanquet et al., 2022). Second, the genetic diversification of serotype 9N. In the pre-pandemic and pandemic period, there was found exclusively GPSC16-ST66, but post-pandemic it diversified in different and new GPSCs: GPSC16-ST66, GPSC699-ST19827 and GPSC137-ST3982. This clonal diversification may reflect selective pressures or transmission changes following the pandemic. Third, the resurgence of 12F serotype linked to a MDR clone within the same GPSC26 and a new ST (from ST989 to ST3377). This new lineage originated after a multifragment recombination involving changes in different genome regions including PBPs. Although its current detection has been limited geographically, its identification in our data underscores the importance of maintaining robust genomic surveillance programs. Only 17 samples have been uploaded to PubMLST (pubmlst.org/, accessed June 4th 2025) and 15 to the GPS project (www.pneumogen.net/gps/, accessed June 4th 2025). Furthermore, 14 records appear in both databases, most of which were isolated in Qatar, with no evidence of the presence of this lineage in Europe. Monitoring the spread and characteristics of such lineage is essential, as their emergence may necessitate revisions of current treatment guidelines and vaccine strategies. Further studies are also needed in order to stablish the putative behavior of this lineage regarding opsonophagocytic killing activity and biofilm formation as was observed in other recombinant strains (Aguinagalde et al., 2015) that could condition its invasiveness.

As observed in IPD cases, serotype 3 was also commonly found in SARS-CoV-2 and *S. pneumoniae* co-infection episodes. On the other hand, serotype 11A, which was not predominant in IPD cases, appeared frequently in these co-infected patients. Serotype 3 isolates were associated with two major lineages previously described in Spain, GPSC83-ST1220 and GPSC12-ST180 (Calvo-Silveria et al., 2024). All serotype 11A isolates were β-lactam-resistant and belonged to the GPSC6-ST6521 lineage, a vaccine escape lineage (formerly serotypes 9V and 14) detected in Spain and other European countries (González-Díaz et al., 2020). Similarly, MDR serotype 6C pneumococci were linked to GPSC47-ST386, as reported previously.

A synergistic effect between SARS-CoV-2 and *S. pneumoniae* in lung inflammation has been demonstrated in mice, leading to increased lethality (Kanta Barman et al., 2022). In our study, despite the low frequency of pneumococcal colonization in SARS-CoV-2 patients, co-infection with both microorganisms appears to lead to a worse outcome, particularly in patients not vaccinated against SARS-CoV-2. It should be noted that many of these episodes occurred at the beginning of the pandemic, when SARS-CoV-2 vaccines were not available. Then, our results may reflect the lack of immunity against SARS-CoV-2, the limited knowledge of the pathogen at the time, and the higher number of severe cases treated in the hospital during the first wave, which could have introduced a bias in the data. However, findings from a case series of ICU-admitted patients with IPD and COVID-19 emphasized that vaccination against both pathogens remain the best strategy to mitigate severe disease outcomes (Almeida et al., 2022).

Our study has several strengths and limitations. A major strength in the approach taken was to analyze the clinical association between SARS-CoV-2 and pneumococcus from a single center, which allowed for detailed analysis of both viral and bacterial lineages. This comprehensive perspective provides valuable insights into the dynamics of co-infections and disease progression during and after the pandemic. However, the study exclusively included adults, and we lacked data on pediatric populations, where the impact of RSV and pneumococcus may be more pronounced. Moreover, the low rate of pneumococcal colonization, observed in SARS-CoV-2 infected patients made it difficult to draw conclusions on the direct impact of SARS-CoV-2 on IPD. Finally, we did not have data on previous SARS-CoV-2 infections, which could influence disease severity and susceptibility to pneumococcal infections.

To conclude, the COVID-19 pandemic significantly altered the epidemiology of IPD, with a sharp decline in cases likely driven by NPIs and reduced circulation of respiratory viruses. The low pneumococcal colonization rate observed in SARS-CoV-2-infected patients suggests that SARS-CoV-2 alone may not strongly predispose individuals to pneumococcal disease. However, the resurgence of IPD following the relaxation of NPIs, particularly during periods of increased RSV and influenza circulation, highlights the importance of understanding viral-bacterial associations and maintaining vaccination programs. Continued surveillance of pneumococcal serotypes, colonization dynamics, and emerging lineages remains critical to inform prevention strategies. Further prospective and multicenter studies including other respiratory viruses are needed to provide a broader perspective on the interplay between viral and bacterial infections in the post-pandemic context, especially in adult population.

## Data Availability

The datasets presented in this study can be found in online repositories. The names of the repository/repositories and accession number(s) can be found in the article/**Supplementary Material**.
