# Supplementary material for: SARS-CoV-2 and Streptococcus pneumoniae colonization and disease: an observational study in adults
Source: Front Cell Infect Microbiol. 2025 Jul 18;15:1624521. doi: 10.3389/fcimb.2025.1624521 (PMC12313665; doi:10.3389/fcimb.2025.1624521)
Supplement: Supplementary file 1 [file Table1.docx]

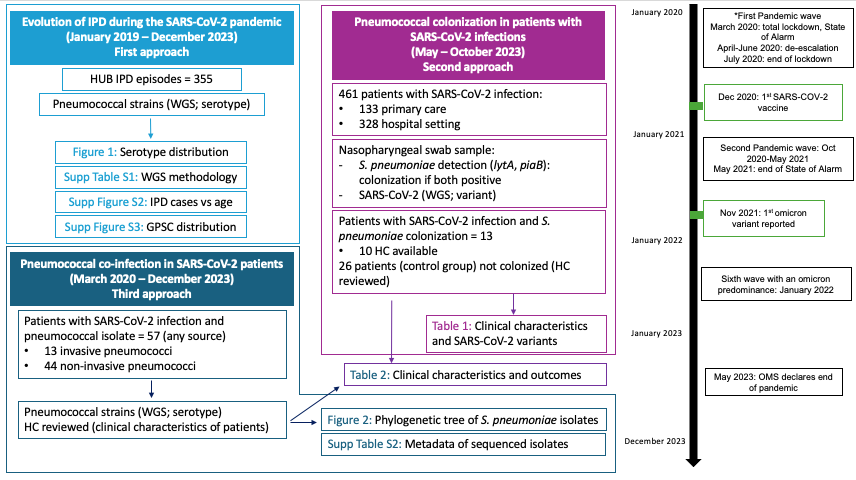


**Supplementary Figure S1. Study design and timeline**.


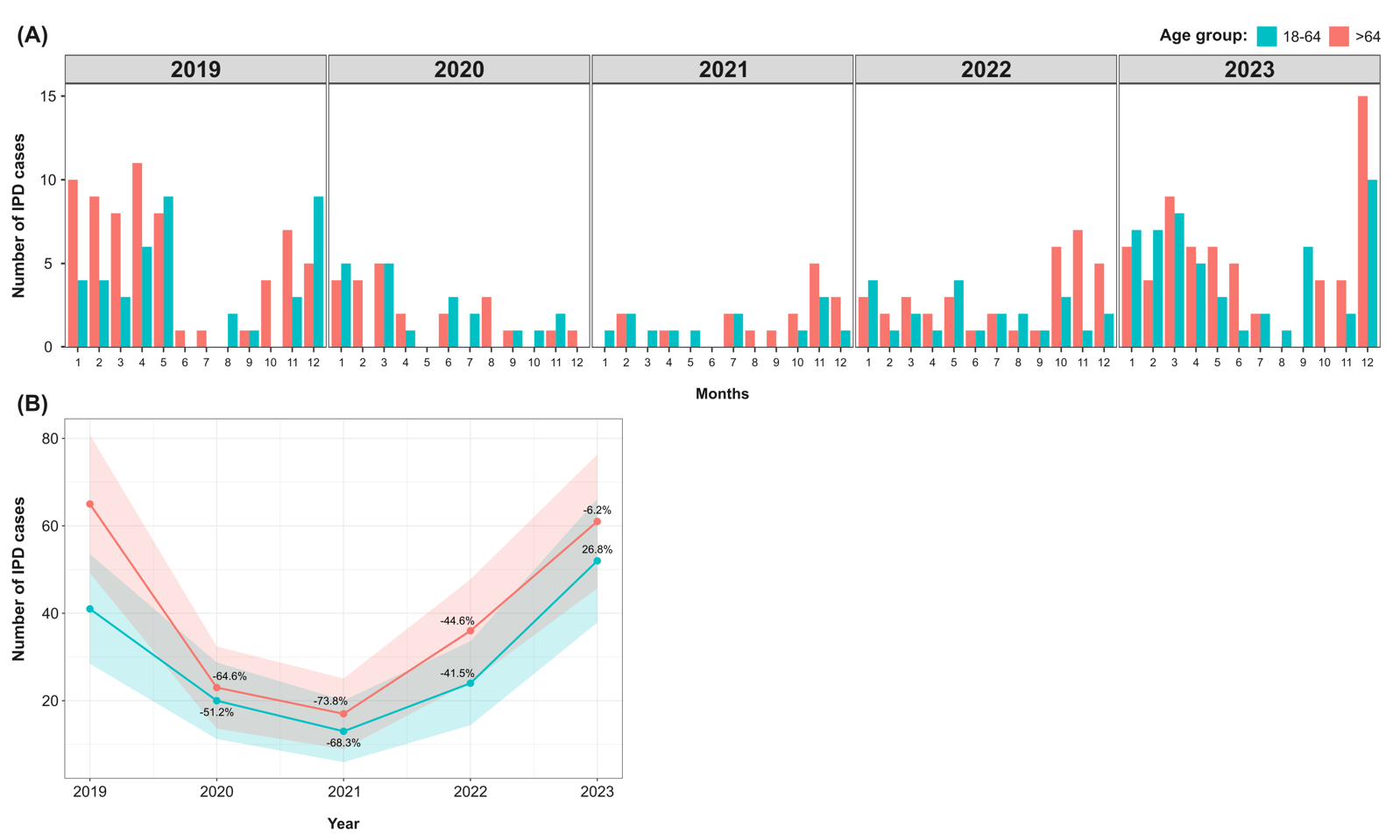


**Supplementary Figure S2. Distribution of IPD cases before and after the COVID-19 pandemic**. Cases of IPD by age group are shown in part (A), while in part (B) the evolution in number of IPD cases by the same age group is shown, in addition to the percentage decrease or increase. The 18-64 group is colored in blue and >64 group is in red.


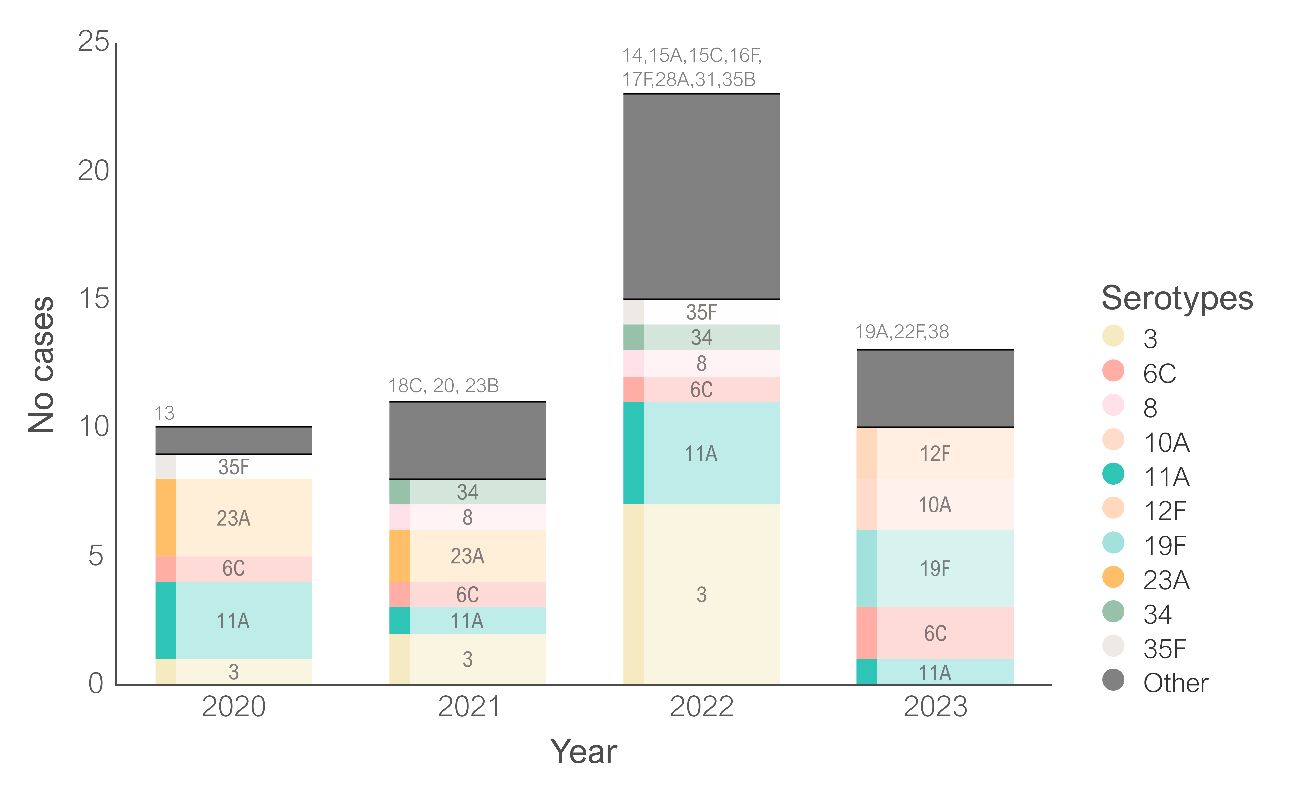


**Supplementary Figure S3. Annual distribution of serotypes in pneumococcal infections among COVID-19 patients.** The total height of each bar reflects the cumulative number of cases for that year.
